# Combination of gemcitabine and docetaxel: a regimen overestimated in refractory metastatic osteosarcoma?

**DOI:** 10.1186/s12885-018-4872-x

**Published:** 2018-10-16

**Authors:** Jie Xu, Wei Guo, Lu Xie

**Affiliations:** Musculoskeletal Tumor Center, Peking University People’s Hospital, 11 Xizhimen South Street, Beijing, China

**Keywords:** Gemcitabine, Docetaxel, Osteosarcoma

## Abstract

**Background:**

The combination of gemcitabine and docetaxel (GT) has been demonstrated to be effective against various types of solid tumors, including sarcoma. However, the regimen has not been confirmed in large, well-designed clinical trials in refractory metastatic osteosarcoma.

**Methods:**

We retrospectively reviewed the records of patients with refractory metastatic osteosarcoma at Peking University People’s Hospital who were treated with gemcitabine (1000 mg/m^2^) intravenously (IV) on Day 1 and Day 8, and docetaxel (75 mg/m^2^) IV on Day 8, repeated every 21 days.

**Results:**

A total of 52 patients with a median age of 18.4 years were treated with GT at the Peking University People’s Hospital from August 2012 to August 2017. A total of 174 courses were administered. Only five patients with pulmonary metastasis achieved a best response of stable disease (SD), while all other patients had progressive disease. The result was disappointing with an ORR of 0%, a DCR of 9.6%, and a median DOR of 3.5 months. Grade 3 or 4 toxicities were observed in 69 (39.7%) courses and in 28 (53.8%) patients, most of which were myelosuppression, especially thrombocytopenia. No fatal adverse effect (AE) was found.

**Conclusion:**

The combination of gemcitabine and docetaxel (GT) as a salvage regimen is well-tolerated but not as effective as expected in refractory metastatic osteosarcoma. This report highlights the need for the development of new approaches with higher activity in these patients.

## Background

Following the implementation of chemotherapy in the 1970s, the treatment of osteosarcoma (OS) has made important progress. However, survival rates continue to be unsatisfactory in the refractory metastatic setting [1].Treatment of these patients is usually difficult and disappointing [2–5].

The first line chemotherapy regimens for patients with OS are designed typically based on four drugs, namely high-dose methotrexate (HDMTX), doxorubicin, cisplatin and ifosfamide [6]. These agents have been incorporated into various chemotherapy protocols. However, OS can develop resistance to conventional agents, resulting in tumor progression or relapse. Local treatment methods such as surgical resection and sometimes radiotherapy are useful in these patients, but unfortunately not able to stop widespread metastases [2]. Once metastasis has occurred and patients have shown refractory to conventional agents, none of current salvage treatments has provided satisfactory results to significantly prolong overall survival [1, 3, 6, 7].

Gemcitabine hydrochloride is a pyrimidine nucleoside analog. Gemcitabine is able to inhibit DNA replication through two different mechanisms: inhibiting DNA synthesis and obstructing repair mechanisms [8]. Docetaxel is a semisynthetic analog of paclitaxel. Docetaxel causes cell cycle arrest and induces apoptosis by promoting microtubule assembly and inhibiting their disassembly. The combination of gemcitabine and docetaxel were initially studied due to their different mechanisms of action and their partially non-overlapping toxicity. Synergistic antitumor activity of the combination of gemcitabine and docetaxel (GT) has been demonstrated in several in vitro studies, including OS [9].

The GT regimen has been used as salvage therapy in several soft tissue sarcoma (STS). An initial clinical study evaluating the efficacy of GT demonstrated a response rate of 53% in 34 adult patients with leiomyosarcoma [10]. A subsequent retrospective study of 35 patients with bone sarcoma and STS treated with GT had an objective response rate (ORR) of 43%, but they had several different tumor types [9]. Only a few studies have reported efficacy and toxicity in OS, including several retrospective [11–17] and one prospective [18] clinical trials. Unfortunately, the results of these studies were controversial and the number of patients was relatively small, ranging from 4 to 35.

Based on the encouraging results in sarcoma, and due to a lack of effective salvage regimens for OS, we have applied GT to patients with refractory metastatic OS at Peking University People’s Hospital from the year of 2012. In this study, we retrospectively reviewed the records of patients treated with GT, with the following purposes: (1) To establish whether the GT regimen is effective in refractory metastatic OS, including ORR, DCR and DOR. (2) To examine the tolerability of GT regimen in heavily treated patients with refractory metastatic OS.

## Methods

### Eligibility

We retrospectively reviewed the patients treated with GT at Peking University People’s Hospital from August 2010 to August 2017. Information about their treatment courses were obtained from the pharmacy medical records in the hospital. Patients were selected according to the following criteria: (1) high-grade OS confirmed histologically; (2) Disease progression was confirmed during the first line treatment with 4-drug protocols consisting of doxorubicin, cisplatin, high-dose methotrexate and ifosfamide (more than 3 months from the initiation of the first line chemotherapy); (3) primary or secondary metastatic disease; (4) received more than 2 courses of the GT regimen; (5) no concurrent treatment was given while on the GT regimen; (6) follow-up information and evaluation after chemotherapy were available.

### Regimen

Gemcitabine (1000 mg/m^2^) was given intravenously (IV) over 90 min on day 1 and 8. Ondansetron (16 mg) was administered prior to initiation of chemotherapy on day 1 and 8. Docetaxel (75 mg/m^2^) was given IV on day 8 over 60 min after gemcitabine. To minimize the severity and incidence of hypersensitivity and the fluid retention associated with docetaxel, dexamethasone treatment was given daily from day 7 to day 9. Each cycle was 21 days. Cycles of chemotherapy were administered until off study criteria were met. Myeloid growth factor support between cycles was given when hematologic toxicity was observed.

### Assessment of toxicity

The toxicity associated with chemotherapy was documented for each cycle according to the National Cancer Institute (NCI) Common Terminology Criteria for Adverse Events (CTCAE version 4.0) [19]. For the patients with unacceptable toxicity, treatment was postponed for up to 42 days, initiated at day 1 of any cycle to allow recovery from toxicity until grade 3/4 symptoms had been resolved. Subsequently, the dosage of GT was resumed at 75% of the previous one. Any patient requiring > 42 days recovery time or > 2 reductions due to toxicity was to be withdrawn from the study.

### Assessment of efficacy

According to the regular protocol for patients with refractory sarcoma in our hospital, the baseline assessment included chest computed tomography (CT, with each layer ≤5 mm) and bone scan or [18^F^]2-fluoro-2-deoxy-D-glucose-positron emission tomography (FDG-PET). If the patients had lesions other than lung metastasis, CT and/or magnetic resonance imaging (MRI) of those lesions was required. Patient follow-up included a chest CT, a CT scan and/or MRI of the baseline lesion every 2 months, and radionuclide bone scans or PET/CT every 6 months. Response to GT therapy was assessed by the RECIST 1.1 criteria [20]. A patient with the outcome of partial response (PR), complete response (CR) or progressive disease (PD) at any stage was scored as having that overall outcome, a patient with the outcome of stable disease (SD) was re-evaluated after two subsequent cycles of therapy. The ORR was defined as the percentage of patients experiencing a CR or PR. The DCR was defined as the percentage of CR, PR or SD. The DOR was defined as the time interval from the initial of treatment to the point of PD in patients who were previously scored as CR, PR or SD. Treatment with GT was to be stopped in case of life-threatening toxicity or progression of the disease. In such cases, patients were encouraged to take part in other clinical trials with targeted therapy, surgery or definitive radiotherapy if possible. Progression-free survival was analyzed using the Kaplan-Meier Method.

## Results

### Patients characteristics

A total of 52 patients and 174 treatment courses were identified. The characteristics of the patients included in this study are summarized in Table 1. The median age of the patients was 18.4 years (range 8–47 years). Among the patients, 47 (90.4%) had a primary lesion in the extremities, while only five patients had primary lesions in the axial skeleton, with two in the sacrum, one in the lumbar region and the other two in the pelvic region. The Eastern Cooperative Oncology Group (ECOG) score in these patients was relatively high, with only 35 (67.3%) of them having a score of less than 2. At the time of the initial diagnosis, 36 (69.2%) patients had localized disease. Before the GT treatment, 40 (77.0%) patients had metastatic lesions in lung, 4 (7.7%) in bone, and 8 (15.4%) in multiple organs. All patients had previously received chemotherapy of 4-drug protocol, and most of them (44/52, 84.6%) were heavy-treated, with a chemotherapy period of more than 6 months, while eight patients had received prior radiation therapy and 48 had undergone previous surgery. All these patients were classified as conventional OS.

**Table 1:** patient characteristics for refractory metastatic osteosarcoma

Patient characteristics	*N* = 52	%
Gender		
Male	29	55.8
Female	23	44.2
Age		
8–14	9	17.3
14–20	22	42.3
> 20	21	40.4
Location of primary lesion		
Extremities	47	90.4
Axial bone	5	9.6
ECOG score		
0–2	35	67.3
> 2	17	32.7
Stage at initial diagnosis		
Localized	36	69.2
Metastatic	16	30.8
Location of metastatic lesions		
Lung	40	77.0
Bone	4	7.7
Multiple organ metastasis	8	15.4
Previous therapies		
Chemotherapy	52	100.0
6 months or more	44	84.6
Less than 6 months	8	15.4
Radiation therapy	8	15.4
Surgery	48	92.3

### Response and survival

**Fig. 1:**
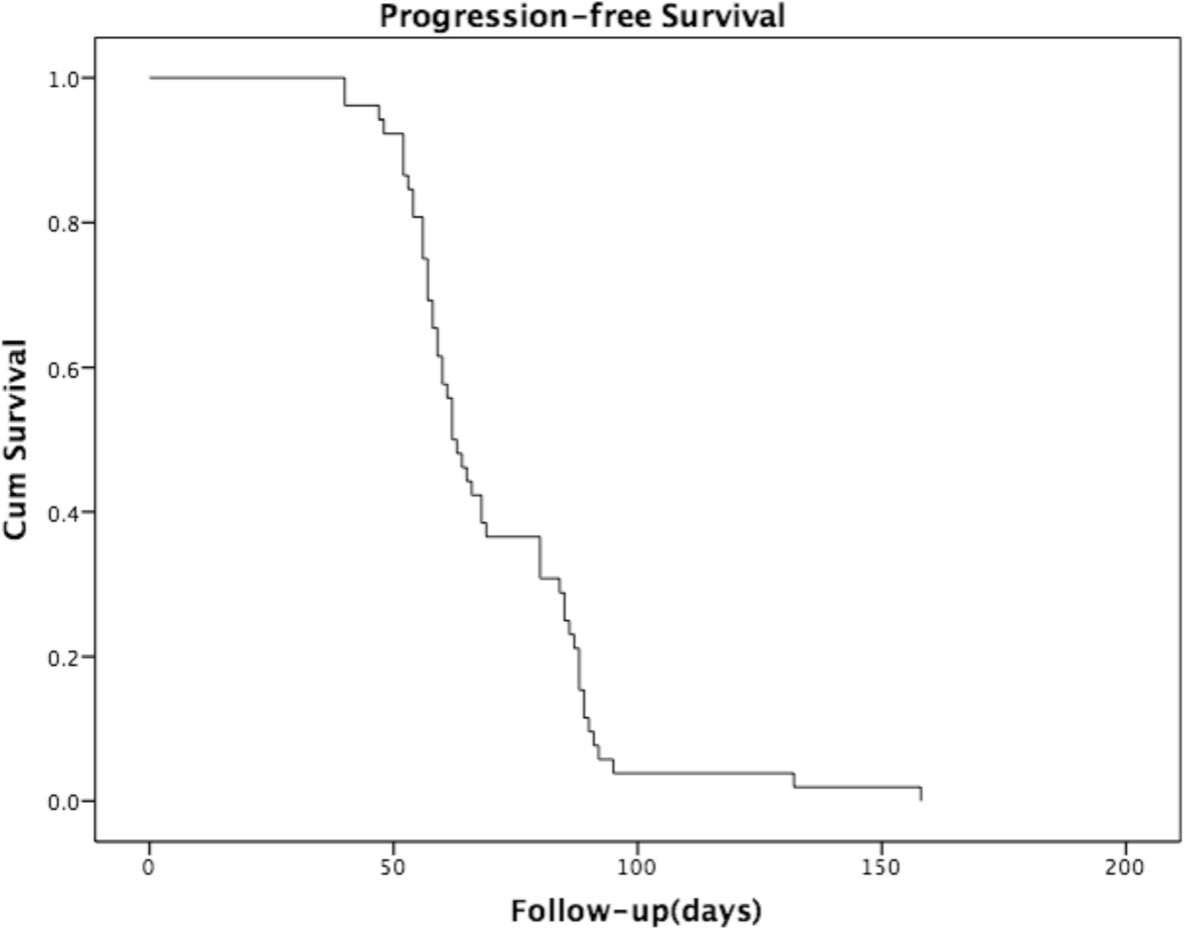
The progression-free survival of patients treated by GT regimen

Unfortunately, no PR or CR was confirmed in this study. The ORR for the confirmed responses according to RECIST1.1 guidelines was 0%. Only five patients with pulmonary metastasis were confirmed as SD. No PR or CR was observed in patients with extrapulmonary lesions, including recurrent lesions and extrapulmonary metastatic lesions. The disease control rate (DCR) was 9.6% (Table 2). Three of the patients had received four courses of GT before PD was observed. Two patients had received six courses of GT in 5.1 months. The median duration of response (DOR) was 3.5 (range 2.0–5.7) months. All the other 47 patients had shown an outcome of PD, in which one patient with prior pulmonary metastasis died due to the rupture of a new onset intracranial lesion after the first course of GT. For patients who experienced PD at first or in the subsequent evaluations, enlargement of the primary lesions was the most common reason (48.1%). Additionally, 23.1% patients showed new lesions while baseline lesions remained stable. Also, 28.8% patients experienced both new lesions and enlargement of baseline lesions. The progression-free survival curve is shown in Fig. 1.

**Table 2:** Response to GT Regimen in 52 patients by RECIST

	Number of patients	%	Mean cycles
CR	0	0	–
PR	0	0	–
SD	5	9.6%	4.8
PD	47	90.4%	3.2
Reason for PD			
New lesions	12	23.1%	–
Lesions enlargement	25	48.1%	–
Both	15	28.8%	–

### Toxicity

Grade 3 or 4 toxicities were observed in 39.7% courses and in 53.8% of the patients. Myelosuppression, especially thrombocytopenia, was the most common toxicity observed. No fatal adverse effect (AE) occurred. The Grade 3 and 4 toxicities observed in the 174 courses of the GT regimen and 52 patients are summarized in Table 3. Neutropenia was found in 55 (31.6%) courses and in 22 (42.3%) patients. Thrombocytopenia was found in 66 (37.9%) courses and in 28 (53.8%) patients. Anemia was found in 52 (29.9%) courses and in 16 (30.8%) patients. Other Grade 3 or 4 toxicities were found in the following aspects: (1) gastrointestinal disorders, including nausea, vomiting, diarrhea and oral mucositis; (2) hepatic disorder, characterized by a transient elevation of alanine transaminase (ALT) or aspartate transaminase (AST); (3) metabolism and nutrition disorders, including hypokalemia, hypocalcemia and hyperglycemia in one patient with prior diabetes. All incidences of Grade 3 or 4 toxicities were less than 5% excluding blood and lymphatic system disorder.

**Table 3:** Grade 3 and 4 Toxicities observed in 52 patients (174 courses) according to CTCAE 4.0

Toxicity	Grade 3		Grade 4	
	events (%)	Patients (%)	events (%)	Patients (%)
Blood and lymphatic system disorders				
Bone marrow hypocellular				
Neutropenia	55 (31.6)	22 (42.3)	0	0
Thrombocytopenia	61 (35.1)	24 (46.2)	5 (2.9)	4 (7.7)
Anemia	52 (29.9)	16 (30.8)	0	0
Febrile neutropenia	3 (1.7)	3 (5.8)	0	0
Gastrointestinal disorders				
Nausea	2 (1.1)	2 (3.8)	0	0
Vomiting	2 (1.1)	2 (3.8)	0	0
Diarrhea	1 (0.6)	1 (1.9)	0	0
Mucositis oral	1 (0.6)	1 (1.9)	0	0
Hepatic disorders				
Elevated ALT/AST	5 (2.9)	4 (7.7)	0	0
Metabolism and nutrition disorders				
Hypokalemia	2 (1.1)	2 (3.8)	0	0
Hypocalcemia	1 (0.6)	2 (3.8)	0	0
Hyperglycemia	4 (2.3)	2 (3.8)	0	0

## Discussion

The GT has a broad spectrum of clinical activity in patients with carcinoma and sarcoma. Research on leiomyosarcoma encouraged the use of GT in sarcoma. In 2002 Hensley reported a retrospective study of 34 leiomyosarcoma with an ORR of 53% [10]. The French Sarcoma Group reported in 2006 an ORR of 24% in 133 STS including leiomyosarcoma [21]. Gemcitabine as a single drug treatment was reported as an effective agent in angiosarcoma by the Italian Rare Cancer Network, with an ORR of 68% [22]. Previous studies have demonstrated that the regimen is well tolerated. However, the prognosis of refractory metastatic OS was disappointing. Doctors have tried to apply the same regimen in this set of patients. Unfortunately, the results of the studies using the same dosage and schedule in OS as that in STS were controversial and the number of patients was relatively small, ranging from 4 to 35 [11–18, 27, 28]. We used the GT in refractory metastatic OS from 2012 to 2017 and used the collected data to investigate the value of GT as salvage therapy. However, in this retrospective study, we did not find evidence to demonstrate that it as an active regimen in OS, with a ORR of 0%, especially in patients with extrapulmonary lesions, since they all experienced disease progression during the GT treatment. In addition, in our study, most patients (48.1%) were evaluated as PD due to the enlargement of baseline lesions. In seven patients with oligometastases who were recorded as PD after GT treatment, their local treatment, such as surgical resection or definitive radiotherapy, was administered based on a multidisciplinary discussion. Four of them had developed secondary metastasis during follow-up (mean 6.4 months, range 3.2–14.5 months), while the other three patients remained disease-free at the latest follow-up. This reminds us that local treatment remains essential for patients with resectable lesions.

This study has several limitations. First, as in most of other reports, our study was retrospective, and most of the patients were heavily treated with various modalities including surgery, radiation, and biological agents, making the interpretation of our data difficult. To guarantee the uniformity of the data, the inclusion criteria were designed and rigidly implemented. The pharmacy medical records of these 52 patients were separately reviewed by two different doctors. Second, overall survival was not investigated in our study. Most of the 52 patients showed PD after the GT regimen, and various salvage therapies were given afterwards, including targeted therapy, radiotherapy, palliative surgery and symptomatic and supportive treatment. The following treatments were considered to have an impact on the overall survival of each patient. In this study, however, we just focused on the GT regimen and overall survival was not adequate to address the activity of a certain regimen.

In previous studies, GT was used as a second-line chemotherapy and response data was reported in ten of them [11–18, 23, 24, 27, 28]. These studies are reviewed in Table 4. The reported ORRs ranged from 0 to 30%, while the DCR ranged from 22.3 to 75%. All of these studies were retrospective, except for a prospective study conducted by Fox E. et al. that was suspended due to poor activity, in which no CR and only one PR was met in fourteen patients [18]. The tumor response rate observed in our study, which was similar to that of Fox et al. [18]. was not as promising as others. The following reasons should be considered to explain the poor outcome. First, in previous studies GT was often used as adjuvant therapy concurrently with or prior to local treatment, which was likely to overestimate the activity of chemotherapy itself. In 2006, Lee J.A. et al. reported a retrospective study including 53 patients with OS using the GT regimen as adjuvant (*n* = 25) or palliative chemotherapy (*n* = 28). In this study all patients who showed a response, both PR or complete metabolic response (CMR), according to PET/CT, had concurrently received local treatment, such as radiotherapy or surgical resection of metastatic lesions. For patients that received GT as palliative treatment alone, only one patient was evaluated as SD, while the other fourteen patients were evaluated as PD. In our study, patients were treated with GT as a single treatment without any concurrent therapy. This should be considered when drawing conclusions. Second, 3/10 [12, 13, 17], 2/10 [15, 24] and [27, 28] of the previous studies reviewed in Table 4 were reported from the same hospital or group at the same time. Thus, the duplication of data should be considered in the analysis. Finally, when comparing with other studies in which both relapsed and refractory patients were included (Table 4), only refractory cases were included in our study. As shown in previous studies, late relapse favorably impacts outcome after relapse [7, 29]. We assumed that the more progressive character in this set of patients who were resistant to first-line chemotherapy was another important reason for the poor response rate. Also, any generalization outside the specific subset of patients studied should be carefully omitted.

**Table 4:** Summary of studies of gemcitabine-docetaxel therapy in osteosarcoma

Author	Journal	Center	Year	No.	ORR	DCR
Fariba Navid [11]	Cancer	St. Jude Children’s Research Hospital	2008	10	3 (30%)	4 (40%)
Yasmin Gosiengfiao [23]	J Pediatr Hematol Oncol	Children’s Memorial Hospital	2012	4	1 (25%)	3 (75%)
Louis Rapkin [14]	Pediatr Blood Cancer	Emory University	2012	5	0	3 (60%)
Elizabeth Fox [18]	The Oncologist	MD Anderson Cancer Center	2012	14	1 (7%)	NA^a^
Weixiang Qi [17]	Jpn J Clin Oncol	Shanghai Jiaotong University	2012	18	1 (5.6%)	4 (22.3%)
Aina He [13]	Int J Clin Oncol	Shanghai Jiaotong University	2013	23	3 (13%)	10 (47.8%)
Wen Xi Yu [12]	Oncology Letters	Shanghai Jiaotong University	2014	21	2 (9.5%)	6 (28.5%)
Bong Sup Song [15]	Pediatr Blood Cancer	Korea Cancer Center Hospital	2014	17	2 (11.8%)	7 (41.2%)
E. Palmerini [16]	BMC Cancer	Instituto Ortopedico Rizzoli	2016	35	6 (17.1%)	20 (57.1%)
Lee JA [24]	Pediatr Blood Cancer	Korea Cancer Center Hospital	2016	53^b^	5 (14.3%)	10 (28.6%)
Tanaka [27]	World J Surg Oncol	Japan Clinical Oncology Group	2016	17 (134)^c^	0	7 (41.2%)
Takahashi [28]	Plos One	Tohoku University Hospital	2017	5 (42)^d^	0	4 (80%)

^a^NA not available

^b^Response datas were available in 35 patients

^c^This study included 134 patients with bone and soft tissue sarcoma, during which 17 (12.7%) patients were diagnosed with osteosarcoma

^d^This study included 42 patients with bone and soft tissue sarcoma, during which 5 (11.9%) patients were diagnosed with osteosarcoma

The toxicity of the GT regimen was acceptable in the current study. Similar to previous reports in OS, Ewing sarcoma and STS [10, 21, 26], the most common grade 3/4 AE related to the GT therapy in our study was blood and lymphatic system disorders, especially neutropenia and thrombocytopenia. Grades 3 and 4 electrolyte abnormalities and transient elevations in ALT/AST were also observed, but less commonly (< 5%). Other common AEs related to gemcitabine and docetaxel, such as rushes, fluid retention and weight gain were not observed in this study. Also, no hypersensitive reaction was found. As patients in our study were already heavily-treated before GT administration, one third of whom had a ECOG performance score of more than 2, the toxicity of this regimen was relatively mild compared with other second-line regimen in OS, such as IE, CE and CT [25].

Recently, small molecule anti-angiogenesis tyrosine kinase inhibitors (TKIs), such as sorafenib, have exhibited more promising potential compared to other target therapies in OS patients [30, 31]. which, in a sense, have become a breakthrough second-line therapy for OS. We have also changed our strategy in these rapidly exacerbating patients and conducted a clinical trial to determine the efficacy of apatinib, an inhibitor for VEGFR-2, in OS. Based on this result, the GT should only be recommended as salvage therapy in frail patients nowadays.

## Conclusions

The GT is recommended in several guidelines as a second-line therapy in OS. However, our study has demonstrated the low tumor response rate in this palliative set of patients. Although this regimen is well tolerated, the disappointing activity prevents us from using it as the salvage therapy in the future. We wonder whether the effectiveness of this regimen is overestimated in the treatment of refractory OS. More effective regimen should be considered and future research should be directed toward more promising agents. Based on the current results of clinical trials in OS, anti-angiogenesis therapy could be a better choice for refractory patients.

## Abbreviations

AE: Adverse effect
CE: Cyclophosphamide-Etoposide
CR: Complete response
CT: Computed Tomography
CTCAE: National Cancer Institute Common Terminology Criteria for Adverse Events
DCR: Disease control rate
DOR: Duration of response
FDG-PET: [18^F^]2-fluoro-2-deoxy-D-glucose-positron emission tomography
GT: Gemcitabine and Docetaxel
HDMTX: High-dose methotrexate
IE: Ifosfamide-Etoposide
IV: Intravenous
MRI: Magnetic Resonance Imaging
NCI: National Cancer Institute
ORR: Objective response rate
OS: Osteosarcoma
PD: Progressive disease
PR: Partial response
